# Longitudinal dataset of human-building interactions in U.S. offices

**DOI:** 10.1038/s41597-019-0273-5

**Published:** 2019-11-26

**Authors:** Jared Langevin

**Affiliations:** Building Technology and Urban Systems Division, Lawrence Berkeley National Laboratory, Berkeley, California USA

**Keywords:** Energy efficiency, Decision making, Energy and behaviour

## Abstract

Adaptive interactions between building occupants and their surrounding environments affect both energy use and environmental quality, as demonstrated by a large body of modeling research that quantifies the impacts of occupant behavior on building operations. Yet, available occupant field data are insufficient to explore the mechanisms that drive this interaction. This paper introduces data from a one year study of 24 U.S. office occupants that recorded a comprehensive set of possible exogenous and endogenous drivers of personal comfort and behavior over time. The longitudinal data collection protocol merges individual thermal comfort, preference, and behavior information from online daily surveys with datalogger readings of occupants’ local thermal environments and control states, yielding 2503 survey responses alongside tens of thousands of concurrent behavior and environment measurements. These data have been used to uncover links between the built environment, personal variables, and adaptive actions, and the data contribute to international research collaborations focused on understanding the human-building interaction.

## Abstract (summary)

**Table:** 

Measurement(s)	behavior • humidity • Occupancy • degree of illumination • atmospheric wind speed • carbon dioxide • Demographics • temperature of air
Technology Type(s)	Thermostat Device • Survey • sensor
Sample Characteristic - Organism	Homo sapiens
Sample Characteristic - Environment	office building
Sample Characteristic - Location	United States of America

Machine-accessible metadata file describing the reported data: 10.6084/m9.figshare.9791435

## Background & Summary

Humans interact with the built environment in a variety of ways that contribute to both building energy use and environmental quality and thus warrant significant attention in the building design, operation, and retrofit processes. Occupants’ thermally adaptive behaviors, for example – adjusting thermostats and clothing, opening and closing windows and doors, operating personal heating and cooling devices – are strongly tied to space heating and cooling loads, which in 2018 comprised 55% and 30% of total site energy consumed in residential and commercial buildings in the United States (U.S.), respectively^1^. These behaviors also modify key thermal comfort predictors like air temperature, air velocity and clothing insulation level^2^. In recent years, an occupant behavior research community has formed around a collection of studies that quantify the magnitude of occupant behavior’s influence on energy use and comfort^3^; the significant impacts reported in these studies have positioned behavior as a key topic of built environment research and related energy policy (e.g.^4–8^). In parallel, a new paradigm of occupant-centric building control has emerged that tunes centralized building operation strategies to real-time feedback on occupant presence, comfort and preferences^9–11^.

While the importance of the human-building interaction is well established, however, the mechanisms that drive this interaction remain largely undetermined. Improving the understanding of these mechanisms requires the collection of longitudinal data, which allow one to observe occupant comfort and adaptive behavior as they evolve together across the day and season^12^. Multiple longitudinal studies have been conducted in Europe across the past two decades, beginning with the Smart Controls and Thermal Comfort (SCATs) project^13^, which tracked thermal comfort, preference, and related behavioral adaptations from 1997–2000 in offices from twenty-five buildings across Europe and first developed models of the probability of a range of adaptive behaviors occurring over time. Subsequent studies have built upon the SCATS data collection approach, examining long-term comfort, window opening, occupancy, and environmental conditions in private and open offices in the United Kingdom^14,15^, Germany^16^, and Switzerland^17^, and using the collected data to develop regression-based models of occupant window interactions.

Such previous studies serve as important precedents for long-term data collection on human-building interactions; however, longitudinal measurements of building occupants are time-consuming and expensive to carry out, and publicly available comfort and behavior data remain limited in their coverage of certain adaptive actions, building types and climates. Few existing studies examine thermal behaviors in air-conditioned buildings, for example, or in buildings in climates with large seasonal variations. Moreover, existing studies rarely examine action hierarchies across several possible thermal behaviors, or the relationship between behavior diversity across an occupant population and individual-level thermal preferences. New longitudinal datasets that address such shortcomings are needed to improve the understanding of interactions between occupants and the built environment.

This paper introduces longitudinal data from a one-year study of occupant thermal comfort and several related behavioral adaptations in an air-conditioned office setting in the U.S. Offices were chosen as the context for the research because of their prevalence in the existing literature on occupant behavior data collection and because they are responsible for the most energy use of any single commercial building type in the U.S.^18^. The study develops a longitudinal protocol based in part on the data collection and analysis approaches of previous long-term occupant studies, adding new items that reflect the theoretical underpinnings of Humphrey’s adaptive principle^19^ and related theories of personal control from the psychology literature^20^. The primary objective of the data collection approach was to record a comprehensive range of exogenous and endogenous factors that may drive personal comfort and behavior outcomes over time, where the latter have not been explored in previous longitudinal studies of occupant behavior despite their suggested importance in previous behavior research^21–24^.

**Fig. 1:**
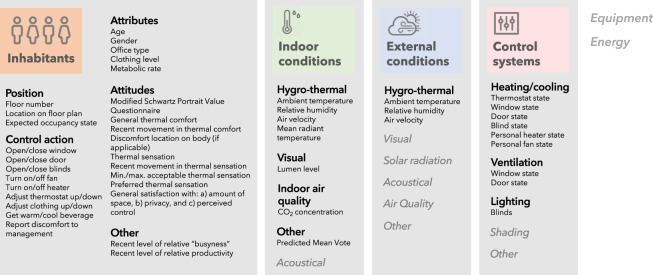
Overview of the monitored building, environment, and occupant variables. Variables are organized according to the data categories and sub-categories in the ontology of Mahdavi and Taheri^25^. Italicized categories or sub-categories were not addressed by the current study.

The longitudinal dataset includes a wide range of variables covering four of the six primary building performance measurement categories identified in the ontology developed by Mahdavi and Taheri^25^, as shown in Fig. 1. For the category of *inhabitants*, the data include information on occupant position in the building, available control actions, personal attributes and attitudes collected from online surveys. Datalogger measurements of *indoor conditions* span the hygro-thermal, visual and indoor air quality sub-categories, while concurrent measurements of *outdoor conditions* include the hygro-thermal sub-category only. Several occupant *control actions* were also measured on a longitudinal basis, using dataloggers where possible and online surveys otherwise; all data streams were merged based on time stamp as described further in the Methods and Data Records sections.

## Methods

Longitudinal data on building occupant behavior, comfort, and environmental conditions were collected between July 2012 and August 2013 at the Friends Center office building in Center City Philadelphia, Pennsylvania, United States. Data collection proceeded in three stages, described further below: (1) semi-structured interviews, (2) site selection and subject recruitment for the longitudinal study, and (3) longitudinal survey and datalogger measurements. Drexel University Institutional Review Board (IRB) approval was received before collecting any of the data reported in this study (protocol #1204001220), and informed consent was obtained from all subjects.

### Semi-structured interviews

Semi-structured interviews identify aspects of behavior that are not yet well known or understood and provide a rich qualitative context for developing and interpreting responses from structured survey instruments^26^. To inform the longitudinal portion of the current study, 32 interviews about thermal comfort and related behaviors were first conducted with office occupants from 7 air-conditioned buildings around the Philadelphia region. The building sample ranged from aging to recently renovated; small to large size; and suburban to urban. Interview responses were recorded and scored using the approach reported in Langevin *et al*.^26^.

### Site selection and subject recruitment for the longitudinal study

The Friends Center office building in downtown Philadelphia was chosen for the full longitudinal study following its inclusion in the semi-structured interviews. At 5200 m^2^ and four floors (one sub-grade), the Friends Center represents a medium-sized office for the region. The brick-faced building, which was constructed in 1972, has a Building Automation System (BAS) following a renovation to LEED Platinum in 2009, and offers many adaptive opportunities to its occupants. Moreover, the building’s interior environment was reported to vary noticeably across the seasons and occasionally within a day, according to the occupant interviews.

Work spaces in the Friends Center include single and shared private offices (enclosed by full height, opaque walls with interior windows), partially open offices (cubicles defined by partial height, opaque partitions), and fully open offices; offices are located at either the core or perimeter of the building floor plan. Perimeter offices are each in close proximity to an exterior window, and the majority of perimeter offices face east, with smaller numbers facing north and west. Spectrally selective, high performance exterior glazing surfaces are offset by a few feet from the brick surface of the building facade, but are otherwise unshaded.

Subject recruitment was initiated through an e-mail message sent to all employees in the Friends Center by its Executive Director. The message outlined the general research approach, the project time period, and remuneration for participants. Included in the e-mail was a link to an online background survey that occupants could fill out to indicate their interest in participating in the full study. The survey was administered via SurveyGizmo and participation was compensated with a $5 gift card. The following question areas were included: (a) demographic information, (b) office characteristics, (c) thermal comfort and preferences, (d) control options, (e) personal values, and f) typical work schedule (arrival, lunch, departure times). A full background survey instrument is available for testing^27^.

The background survey generated a total of 45 occupant responses; from this initial sample, a final sample of 24 occupants was selected for participation in the full longitudinal study using a non-proportionate quota sampling strategy^28^. The strategy looked to achieve at least 1/3 of the final sample (or 8 occupants) in each of a series of key groupings, defined in order of importance by: gender; office type and location; behavioral control options; and comfort/perceived control satisfaction level. Table 1 presents the final occupant sample in terms of these categories.

**Table 1:** Final occupant sample characteristics (total N = 24).

Category	Options	N	% Final Sample
Gender	Male	8	33
	Female	16	67
Office Type	Private	6	25
	Shared Private	3	13
	Cubicle	10	42
	Open Desk	5	21
Office Location	Perimeter	15	62
	Core	9	38
Control Available (Control Used)	Heater	14 (4)	58 (17)
	Fan	15 (5)	63 (21)
	Thermostat	12 (10)	50 (42)
	Windows	17 (12)	71 (50)
	Doors	12 (9)	50 (38)
	Blinds	20 (16)	83 (67)
General Thermal Comfort	≥“Comfortable”	19	79
	<“Comfortable”	5	21
General Perceived Control Satisfaction	≥“Satisfied”	15	62
	<“Satisfied”	9	38

### Longitudinal survey measurements

Over the course of the following year, the final occupant sample participated in a series of subjective and objective measurements of thermal comfort, adaptive behavior, and related items. These measurements were carried via longitudinal online surveys, as well as through parallel datalogger and BAS measurements of the local environment and behavioral actions; the surveying approach is first outlined here.

Longitudinal surveys were distributed online via SurveyGizmo three times daily (morning, mid-day, afternoon) for a two week period in each of the four seasons, with the time of each survey distribution tailored to work schedules that occupants reported on the background survey. At the time of data collection, SurveyGizmo did not have a native function for repeated surveying of the same subjects; accordingly, the Gmail plugin Boomerang was used to send participants survey links at pre-specified daily times. To incentivize participation, occupants were offered a $5 gift card for every full day of surveying completed, in addition to the $5 previously received for participation in the background survey.

**Fig. 2:**
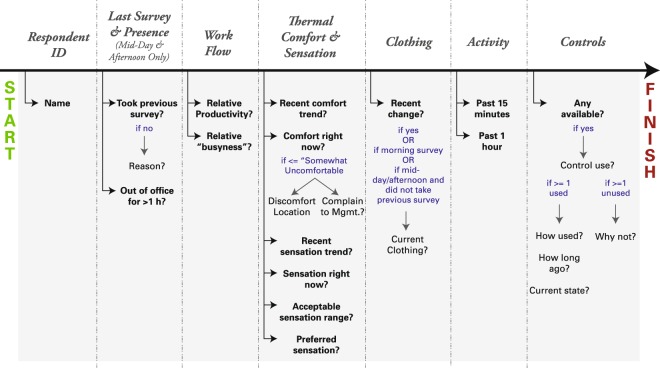
Diagram of question progression on daily online surveys. Survey questions cover the occupant’s recent work flow, current thermal comfort and sensation, current and recent clothing levels, recent activity, and recent control use. Median time to complete the survey was 2.3 minutes across all responses. Adapted with permission from^38^.

The daily survey instruments included questions about recent work flow, current thermal comfort and sensation, current and recent clothing levels, recent activity, and recent control use. Figure 2 diagrams the full progression of daily survey questions, indicating both the general question areas and associated areas of sub-questioning. A daily survey instrument is available for testing^29^.

Many of the daily survey questions shown in Fig. 2 follow a “show when” logic, where their appearance is conditional on a response to a previous question. The use of “show when” logic minimizes unnecessary questioning, keeping survey completion times down and combating possible survey fatigue. Fatigue was also countered through the use of several image response options, including a series of clothing checklist icons, for example.

Once the daily surveys had been filled out for the full two week period, each occupant completed a final survey that asked about the whole of their two week experience. A two week retrospective survey instrument is available for testing^30^. The final survey asked occupants to log any times when they were out of the office for a significant time (e.g., a whole or half day), also querying general comfort and the frequency of behaviors during the previous two week period. The survey also asked occupants to assess the accessibility, time expense, and general clarity of the daily survey instruments. Pairing this feedback with a close examination of the daily survey responses during the given two week period helped inform small revisions to the daily surveys between each seasonal run. Revisions included streamlining the general flow of questions and the addition of short follow-up prompts (for example, on the bodily location of reported thermal discomfort). Revised daily survey instruments were tested through two-day distributions in the time between the full two week seasonal runs to ensure the readiness of these revised instruments for the next two-week implementation.

Overall, the daily surveys generated consistently high response rates (>=85% in each season), yielding a total of 2503 responses from the 24 subjects. The surveys took a median time of just 2.3 minutes to complete and were positively evaluated by occupants. Table 2 summarizes key statistics regarding the performance of the daily survey instruments during each two week seasonal run as well as during the two day test runs in between.

**Table 2:** Summary of daily survey instrument performance by seasonal period.

Variable	Summer	Sum/Fall 2 Day	Fall	Fall/Wint 2 Day	Winter	Spring	Overall
N responses	593	116	570	113	545	566	2503
N links sent	692	126	670	129	642	666	2925
Response rate (%)	86	92	85	88	85	85	86
Median responses/person	26	6	26	5.5	27	26	117
Max questions/survey	10	31	23	28	28	29	—
Median time/survey (mins.)	1.5	4	2.7	2.8	2.4	2.4	2.3
Overall experience^a^	4.6	—	4.2	—	4.6	4.6	4.5
Accessibility^a^	4.7	—	4.5	—	4.8	4.6	4.6
Time consumed^a^	4.7	—	4.4	—	4.5	4.3	4.5
Clarity of questions^a^	4.3	—	4	—	4.3	4.2	4.2

^a^On a scale of 1 = needs major improvement to 5 = very good. Shown is the average of responses.

### Environment and behavior datalogger and building automation system (BAS) measurements

Alongside the longitudinal survey data collection, continuous measurements were made of the weather, the local indoor environment of each occupant’s office, and a subset of the occupants’ behavioral actions. Table 3 summarizes each of these measurements and their observed ranges during the two week surveying periods and across the entire year of the project.

**Table 3:** Summary of environment and behavior measurements across the year.

Variable Type	Variable	N People	Measure Interval	Download Interval	Value Range^a^ (Surveys), Median [IQR]	Value Range^a^ (Whole Year), Median [IQR]
Outdoor Environment	Ambient Temp. (ºC)	—	1 hour	1 month	10 [6, 18]	15 [7, 24]
	Rel. Humidity (%)	—			55 [43, 74]	55 [42, 73]
	Wind Speed (m/s)	—			4.0 [2.7, 5.9]	4.0 [2.7, 5.9]
Indoor Environment	Ambient Temp. (BAS, °C)	24	15 mins. (BAS data), 5 mins. (Other data)	1–2 weeks^b^	23.0 [21.6, 24.0]	23.1 [22.0, 24.0]
	Ambient Temp. (HOBO, °C)	11			22.8 [21.7, 23.7]	22.9 [21.9, 23.7]
	Rel. Humidity (%)	11			30.9 [25.7, 41.3]	38.5 [25.7, 52.9]
	Globe Temp. (ºC)	8			23.1 [22.0, 23.9]	23.0 [22.0, 23.9]
	Air Velocity (m/s)	4			0.027 [0.026, 0.037]	0.027 [0.026, 0.037]
	Illuminance (lm/ft^2^)	4			82.9 [35.5, 137.8]	75.4 [28.0, 130.2]
	CO_2_ (ppm)	3			561.1 [484.1, 656.3]	536.0 [477.4, 637.4]
Behavior	Fan Use (Watts)^c^	4	15 mins. (fans/heaters), On state change (windows)	1–2 weeks^b^ (fans/heaters), 1 month (windows)	—	—
	Heater Use (Watts)^c^	5				
	Window State (Open/Closed)	10				

^a^Restricted to the occupied hours of 7 AM–6 PM. ^b^Read every 1 week during survey times, and every 2 weeks otherwise. ^c^Based on observation of the WattsUp meter readings during the one month test period before the formal start of the study, fan readings between 3 and 125 Watts were marked as “On,” heater readings between 50 and 1500 Watts were marked as “On,” readings of 0 Watts were marked as “Off” for both devices, and any other reading was marked “Faulty”.

Historical hourly weather data (temperature, humidity, and air velocity) were downloaded on a monthly basis from the Weather Analytics service (now Athenium Analytics) for the “Center_City_Philadelphia_580144” grid point.

Regarding interior conditions, ambient temperature was measured for every occupant using the nearest thermostat reading logged by the BAS system at 15 minute intervals. Ambient temperature and relative humidity were also logged at 5 minute intervals for 11 occupants using HOBO U12-013 base sensors (accuracy: +/−0.35 °C temperature, +/−2.5% humidity), as was globe temperature in eight cases (TMC6-HE attachment covered with 40 mm black matte ping pong ball; +/−0.25 °C accuracy), and air velocity in four cases (T-DCI-F900-S-O attachment; +/−0.05 m/s accuracy). Globe temperature sensors were placed on the perimeter and core areas of each floor space, and one air velocity sensor was placed on each floor. Additionally, illuminance and CO_2_ concentration were logged for certain occupants across floors using the HOBO U12-012 sensor (accuracy dependent on wavelength^31^) and the TelAir 7001 CO_2_ sensor (accuracy: greater of +/−50 ppm or 5% of reading), respectively. Sensor calibration was not performed after initial deployment. Further information on the location of each sensor including floor number, perimeter vs. core position within each floor, and office type are provided in the data record.

Datalogger measurements of occupant behavior recorded window adjustments and the use of personal fans and heaters. For windows, HOBO UX-90 state loggers were attached to 10 different windows that occupants in the sample reported using in the background survey. The UX-90 loggers record the time stamp of any state change during the measurement period. For fans and heaters, WattsUp? Pro power meters logged wattage at occupant-designated fan/heater outlets at 15 minute intervals. The WattsUp? meters were installed at the desks of all 9 occupants who reported using a heater or fan at some point throughout the year on the background survey.

**Fig. 3:**
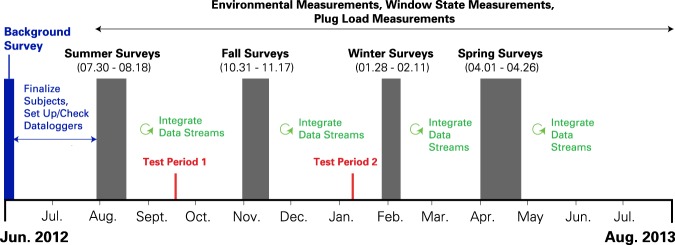
Collection and integration of survey, environmental, and behavior data streams across one year. Each occupant completed 2 weeks of daily surveying within the stated survey time window for each season. Adapted with permission from^38^.

Together with the daily survey responses, the above environment and behavior information comprises six longitudinal data streams (daily surveys, outdoor weather, BAS thermostats, local environment, personal fans and heaters, and windows). Figure 3 provides an overview of the collection and integration of these various data streams. The Data Records section provides additional detail on how each data stream was post-processed and matched with the other data streams.

## Data Records

A series of MATLAB^32^ scripts were used to process and integrate the environmental and behavior data streams listed in Table 3 as well as all survey data. The general post-processing and integration procedure used by these scripts is as follows:

1. Download the raw data at intervals specified in Table 3 (CSV format); survey data were downloaded at the end of the initial background survey period, and at the end of each two day test period and two week surveying period.
2. Remove any missing readings in the data or data points that are determined to represent faulty measurements based on observation of the building at the time of data download (e.g., measurement device was disturbed during measurement period or atypical operational patterns were observed during the measurement period).
3. Organize raw CSV data readings into larger CSV files along the following dimensions:

- Measurement type (BAS, HOBO, weather, heater/fan, window, daily survey).
- Occupant number (1 to 24).
- Measurement variable (e.g., temperature, humidity, fan use, etc.).
- Reading number (sequentially ordered from start to end of the study).

4. Use MATLAB scripts to merge all CSVs from step 3 into a single .txt file for each measurement type, mapping the CSV time stamps in date/time format to serial date numbers using the MATLAB *datenum* function.
5. Use a MATLAB script to merge .txt files from step 4 into a master .txt file that includes all measurement types in one place. In the script, disparate measurements are matched by time step in serial date form; where a certain measurement does not exist for a given time step (e.g., survey data in a time step where there were no surveys), a NaN value is shown. Additionally, a subroutine for calculating the Predicted Mean Vote (PMV) from measured environmental and occupant variables is used; this module follows the routine established in ASHRAE Standard 55^33^ for the PMV calculation.

The dataset that was produced by these steps is made available under an Creative Commons Attribution 4.0 International license through Zenodo^34^. All data are stored in a compressed .txt file (“LANGEVIN_DATA.txt”), which is accompanied by a data legend file in XLSX format (“langevincodebook.xlsx”). The data legend file characterizes each variable column found in the .txt file by type (environment, behavior, etc.), name, data collection source (surveys, dataloggers, etc.), value type (discrete, continuous), description, units, and response levels (for discrete variables).

## Technical Validation

Several measures were taken to ensure the validity of the collected data, following data collection guidance included in the final report for International Energy Agency Annex 66: Definition and Simulation of Occupant Behavior in Buildings^35^. These measures are outlined below.

### Survey preparation phase

Longitudinal survey design was informed by preceding semi-structured interviews with office occupants along with background surveys of each occupant’s demographic characteristics, thermal comfort, and adaptive behavioral tendencies. Information collected during this preparation phase informed the design of longitudinal survey instruments, ensuring that the questions included on these surveys would be correctly interpreted by occupants and relevant to their daily experience of the office environment.

### Encourage high response rates

The inclusion of financial incentives for participants and use of “show-when” logic to minimize survey fatigue yielded a high overall response rate of 86% across the longitudinal surveys (Table 2), ensuring the survey data are not biased towards respondents with higher participation rates.

### Pilot studies

Three small pilot studies were conducted during the course of the full longitudinal study: one in the month preceding the start of the study, during which time all required dataloggers were installed and collecting test data, and two in the periods between the summer and fall and fall and winter daily surveys, during which times improved survey deployments were tested alongside logger data collection. The pilot studies verified that all data collection devices and the online survey platform were functioning properly before each of the two week daily surveying periods was conducted.

### Quality control

Datalogger readings were downloaded regularly (at maximum every 2 weeks) to ensure their proper functionality and, in the case of environmental sensors, to verify that readings had not drifted significantly. Between these download times, sensor operation was visually inspected every few weekdays to ensure the devices were actively recording data and had not been moved or dislodged by occupants.

### Redundancy

In the case of outdoor and indoor temperature, parallel data streams were compared to address internal validity. For outdoor temperature, hourly data points provided by Weather Analytics were spot-checked against concurrent measurements from the nearest weather station (KPHL) on Weather Underground. For indoor temperature, 5 minute interval HOBO temperature measurements were compared against concurrent BAS temperature measurements from the nearest thermostat. Here, summary statistics for whole year BAS temperature readings are within the margin of error of the HOBO temperature readings, indicating a close correspondence between the two measurements. Additionally, state logger measurements of behavior (heaters, fans, windows) were verified against corresponding daily survey responses that reported recent behavioral actions.

### Comparison against expected conditions

One year inter-quartile ranges for each of the environmental variables in Table 3 are consistent with those expected of an air-conditioned U.S. office building with occupants that report high levels of general environmental satisfaction, specifically:

- The range of ambient and globe temperature (22.0 °C to 24.0 °C and 22.0 °C to 23.9 °C, respectively) fall within the approximate range of acceptable operative temperatures in ASHRAE Standard 55^33^ (20.5 °C to 28 °C),
- The range of relative humidity (25.7% to 52.9%) falls below the relative humidity threshold recommended by ASHRAE Standard 62.1^36^ (at or below 65%),
- The range of CO_2_ concentration (477 ppm to 637 ppm) falls below the indoor CO_2_ concentration threshold recommended by ASHRAE Standard 62.1 (below 1000 ppm), and
- The illuminance range (28.0 lm/ft^2^ to 130.2 lm/ft^2^) encapsulates the average value recommended by the IES Lighting Handbook^37^ for commercial offices (40 lm/ft^2^), though the median illuminance level (75.4) is higher than the recommended value, likely reflecting the widespread access to daylighting in the studied building.

## Usage Notes

The longitudinal dataset described herein was first posted on OpenEI in July of 2015; in the years since, several researchers have contacted the author for clarifications on certain aspects of the data. Based on these inquiries, notes to guide further use and interpretation of the data are summarized below.

- When reading in the main .txt file to a program such as R or Python for analysis, variables of interest should be indexed by the associated column number listed in the variable codebook file (“langevincodebook.xlsx”).
- NaNs for certain variables do not generally imply missing data; rather, they indicate that the variable was not being measured for a given occupant at a given time stamp. For example, per Table 2 only four occupants had a direct illuminance measurement at their office location; for the occupants *without* this direct measurement, the illuminance variable will show NaNs across all time steps. Similarly, variables such as current and acceptable thermal sensation will show NaNs for all time steps that fall outside of the two week daily surveying windows.
- The variable “Occupant Number” refers to an occupant ID (e.g., a value between 1 and the 24 occupants who participated in the study); it does not refer to an occupancy (presence/absence) measurement.
- The variable “Occupancy 1” is an expected occupancy (presence/absence) value calculated *across all time steps in the study* based on the occupant’s responses about typical periods of occupancy on the background survey conducted before the start of the longitudinal measurements. The variable “Occupancy 2” is an expected occupancy state calculated *across all time steps that fall under the two week daily surveying window*, based on the time of arrival reported in the daily morning survey and recent departures from the office reported in the daily mid-day and afternoon surveys, as well as periods of prolonged absence reported on the retrospective surveys conducted after each two week period. Occupancy state was not continuously measured with dataloggers in this study.

**Fig. 4:**
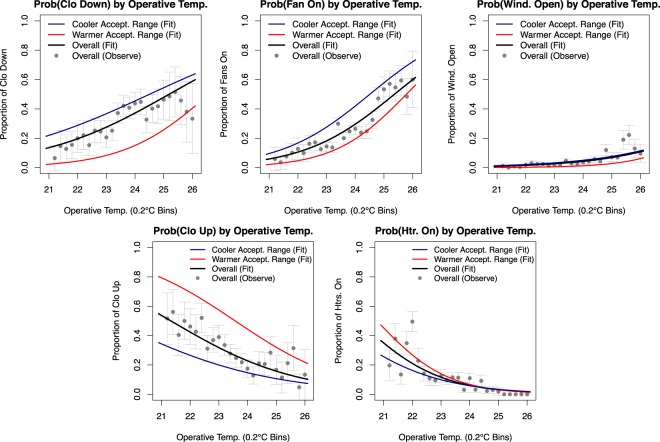
Using daily survey data collected across two week periods in each season of the study and concurrent datalogger measurements of the thermal environment and behavior, relationships between indoor operative temperature and the probability of behavior states are plotted. Logistic regression curves are shown for the total sample of occupants with each behavior available and for each sample grouped by cooler and warmer acceptability range. Gray dots are observed proportions of behavior use in the study with 95% confidence intervals. Increasing probabilities of high clothing insulation, fans turned on, and open windows are observed with increasing operative temperature, while increasing probabilities of heavier clothing and heaters turned on are observed with decreasing operative temperature. Occupants with warmer acceptability ranges are significantly less likely to engage in behaviors that address warm discomfort, while occupants with cooler acceptability ranges are significantly less likely to engage in behaviors that address cool discomfort. Adapted with permission from^38^; see^38^ for details on clothing and acceptability range definitions.

**Fig. 5:**
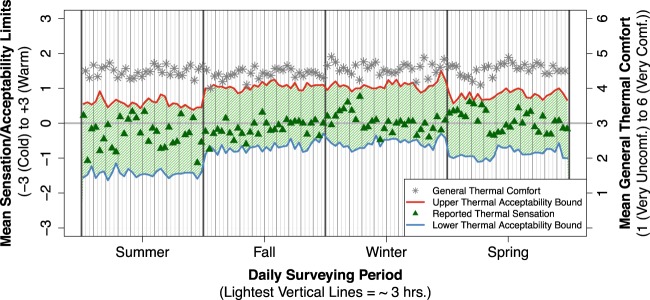
Using daily survey data collected across two week periods in each season of the study, the evolution of mean thermal sensation, acceptability, and comfort responses is plotted. Thermal sensation generally remains within the acceptable ranges, with associated high ratings of general thermal comfort observed across each two week period. Comparing acceptability ranges between the summer/spring and fall/winter periods, a substantial shift in this range is observed in the direction of warmer thermal sensations. Adapted with permission from^38^.

**Fig. 6:**
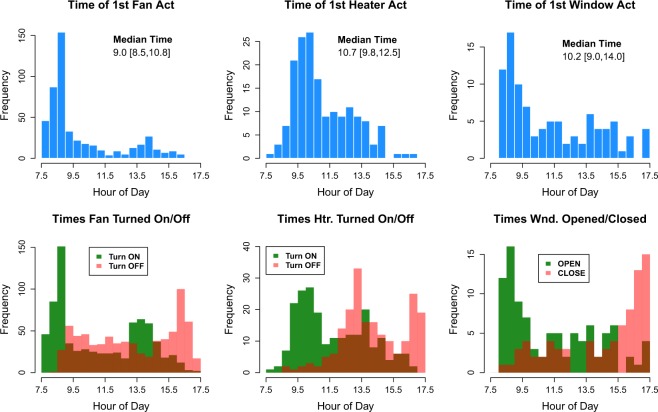
Using fan and heater data collected across the full year of the study, the time of first daily fan, heater, and window action is plotted, along with the times that fans and heaters are turned on and off and windows are open and closed. Fans and windows tend to be turned on/opened upon arrival at the office; while fans tend to be turned off in late morning and on again after returning from lunch, windows appear to remain open until departure from the office. Heater use peaks in the late morning hours, with most heaters turned off by the early afternoon. Adapted with permission from^38^.

Example applications of these longitudinal data in analysis and modeling efforts are found in both the author’s own work and those of other researchers pursuing research on occupant comfort and behavior. Regarding the author’s work, these data were used in^38^ to develop insights on the drivers of behavior (Fig. 4), long-term trends in thermal comfort and acceptability (Fig. 5), and the sequencing of adaptive actions (Fig. 6). The author also used the data to develop and validate an agent-based model of occupant behaviors in^39^, demonstrating the richness of the dataset for studies of either individual- or group-level behaviors in office settings.

Outside of the authors’ own work, several other researchers have used the longitudinal data to explore relationships between the built environment and occupant comfort and behavior, most notably: (1) the dataset was included in the recently developed ASHRAE Database II project^40^, a collection of 81,846 complete sets of objective indoor climatic observations with accompanying “right-here-right-now” subjective evaluations by the building occupants who were exposed to them, which is intended to support diverse inquiries about thermal comfort in field settings, and (2) the data were used in^41^ to develop synthetic profiles of building occupant personal characteristics and comfort preferences that can be used to intialize models of individual-level behaviors (for example, in an agent-based framework). The latter effort continues to be pursued and expanded through the recently approved International Energy Agency Annex 79 on Occupant-Centric Building Design and Operation^42^.

## Data Availability

All MATLAB scripts used to implement the post-processing steps described in the Data Records section are available upon request, as are the raw data files described in steps 1 and 2 (e.g., pre- and post- cleaning). Interested readers should contact the corresponding author for access to the scripts so that further instructions on their use may be provided. Additionally, links to the background survey instrument^27^, daily survey instrument^29^, and retrospective survey instrument conducted after each two week daily surveying period^30^ are available for testing.
